# How fall dormancy benefits alfalfa winter-survival? Physiologic and transcriptomic analyses of dormancy process

**DOI:** 10.1186/s12870-019-1773-3

**Published:** 2019-05-20

**Authors:** Zhi-Ying Liu, Taogetao Baoyin, Xi-Liang Li, Zong-Li Wang

**Affiliations:** 1 Key Laboratory of Grassland Ecology, School of Ecology and Environment, Inner Mongolia University, Hohhot, China; 2 Key Laboratory of Grassland Ecology and Restoration of Ministry of Agriculture, National Forage Improvement Center, Institute of Grassland Research, Chinese Academy of Agricultural Sciences, Hohhot, China; 3 China Animal Health and Epidemiology Center, Qingdao, China

**Keywords:** *Medicago sativa*, Fall dormancy, RNA-Seq, qRT-PCR, Raffinose and amino acids

## Abstract

**Background:**

Fall dormancy and freezing tolerance characterized as two important phenotypic traits, have great effects on productivity and persistence of alfalfa (*Medicago sativa* L.). Despite the fact that one of the most limiting traits for alfalfa freezing tolerance in winter is fall dormancy, the interplay between fall dormancy and cold acclimation processes of alfalfa remains largely unknown. We compared the plant regrowth, winter survival, raffinose and amino acids accumulation, and genome-wide differentially expressed genes of fall-dormant cultivar with non-dormant cultivar under cold acclimation.

**Results:**

Averaged over both years, the non-dormant alfalfa exhibited largely rapid regrowth compared with fall dormant alfalfa after last cutting in autumn, but the winter survival rate of fall dormant alfalfa was about 34-fold higher than that of non-dormant alfalfa. The accumulation of raffinose and amino acids were significantly increased in fall dormant alfalfa, whereas were decreased in non-dormant alfalfa under cold acclimation. Expressions of candidate genes encoding raffinose biosynthesis genes were highly up-regulated in fall dormant alfalfa, but down-regulated in non-dormant alfalfa under cold acclimation. In fall dormant alfalfa, there was a significantly down-regulated expression of candidate genes encoding the glutamine synthase, which is indirectly involved in the proline metabolism. A total of eight significantly differentially expressed transcription factors (TFs) related to CBF and ABRE-BFs were identified. The most up-regulated TFs in fall dormant alfalfa cultivar were ABF4 and DREB1C.

**Conclusions:**

Fall dormant alfalfa drastically increased raffinose and amino acids accumulation under cold acclimation. Raffinose-associated and amino acid-associated genes involved in metabolic pathways were more highly expressed in fall dormant alfalfa than non-dormant alfalfa under cold acclimation. This global survey of transcriptome profiles provides new insights into the interplay between fall dormancy and cold acclimation in alfalfa.

**Electronic supplementary material:**

The online version of this article (10.1186/s12870-019-1773-3) contains supplementary material, which is available to authorized users.

## Background

Plant dormancy is described as the absence of visible growth and it is a flexible adaptive strategy, which is essential for developmental synchronization and survival of woody and other perennial plants in stressful environments [10, 26, 46, 63]. For example, bud dormancy, a case of endodormancy, allows perennial plants of temperate and boreal zones to survive low winter temperatures. But this dormancy has a negative impact on productivity of perennial herbaceous plants worldwide [5]. Thus, understanding the correlation between mechanisms involved in plant dormancy and cold acclimation will provide information which may be utilized by plant breeders to develop new cultivars with improved tolerance to low temperature. Recently, several adaptive strategies to cope with dormancy have evolved in woody and herbaceous plants, including poplar (*Populus* spp.) [51], apple (*Malus×domestica* Borkh) [56], grape (*Vitis vinifera* Linn.) [21], strawberry (*Fragaria×ananassa* Duch.) [60], kiwifruit (Actinidia *spp.*) [54], and *Astragalus scaphoides* and *Silene spaldingii* [52]. These strategies involve physiological and molecular responses, such as carbohydrate and hormonal changes, and changes in DNA methylation patterns. Hundreds of plant genes that contribute to the intricate regulation of dormancy have been reported [13, 20, 38, 58], providing useful underpinnings for further studies of perennial plant dormancy [57].

To date, it has been recognized that the physiological process of cold acclimation is the synthesis of compatible solutes, i.e. raffinose family oligosaccharides (RFOs) and amino acids [15, 17, 19, 36]. Raffinose (one of the RFOs) as a good indicator of freezing tolerance, has accumulated largely under cold stress in many plants, such as *Ajuga reptans*, which is a frost-hardy perennial labiate [3], *Arabidopsis thaliana* [29], vetch (*Vicia villosa*) [31], rice (*Oryza sativa*) [47] and cucumber (*Cucumis sativus*) [49]. The synthesis of raffinose is catalyzed by raffinose synthetase. It has been described that raffinose plays a role in protecting photosystem II of *Arabidopsis* from damage in cold acclimation [29]. Amino acids are also the common solutes that accumulate in many plants when exposed to cold acclimation, such as chrysanthemum [11]. The accumulation of amino acids is mainly induced by related enzyme in the amino acids metabolism pathway, which improves cold tolerance [11].

Alfalfa (*Medicago sativa* L.), the world’s most extensively cultivated perennial forage legume, has a key feature of transition from growth to dormancy in the autumn [7]. After harvesting in late summer or early autumn, different alfalfa varieties show diverse growth speeds, leading to differences in shoot growth height in autumn. This phenomenon is characterized as fall dormancy (FD) [6]. FD rating of alfalfa cultivars can be grouped into 11 FD classes included in three subgroups: fall dormant type (FDT; FD 1–4), semi-dormant type (SDT; FD 5–7), and non-dormant type (NDT; FD 8–11) [2]. Varieties with FD 1 go dormant the earliest, have little or no top growth in the fall and are very winter hardy, and varieties with FD 11 continue to grow and have the highest plant height in the fall, but generally demonstrate poor winter survival [45]. Higher winter survival of FDT cultivars has been related to early initiation of accumulation of soluble sugars and amino acids in roots of alfalfa in autumn [15]. However, the interplay between fall dormancy and cold acclimation processes, and the differences between the FDT and NDT cultivars that might relate to differences in classic cold acclimation processes in alfalfa remain poorly understood.

Here, we used FDT and NDT alfalfa cultivars to examine the correlation between fall dormancy and cold acclimation processes. Winter survival rate, raffinose, and amino acids were measured in FDT cultivar compared with NDT cultivar. A genome-wide transcriptional analysis was conducted to identify differentially expressed genes between FDT and NDT alfalfa cultivars under cold acclimation. Expression changes of a subset of these genes in response to the cold acclimation were confirmed by quantitative Real-Time PCR (qRT-PCR) analysis. The objectives of this study were to (i) examine the correlation between fall dormancy and cold acclimation processes of alfalfa, (ii) explore the differences between FDT and NDT cultivars that might relate to these differences in classic cold acclimation processes.

## Results

### Plant growth and winter survival

**Fig. 1:**
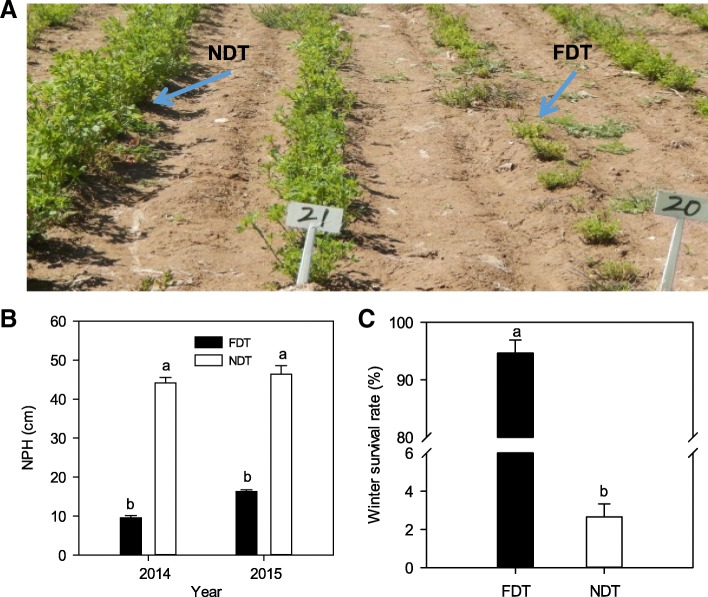
Growing states 25 days after the last cutting (**a**), natural plant height (NPH) in September of both 2014 and 2015 (**b**) and average winter survival rate in two consecutive years (**c**) of alfalfa cultivars selected for contrasting fall dormancy. Fall dormant (FDT) alfalfa (Maverick, FD = 1) is a dormant and winter hardy cultivar, while non-dormant (NDT) alfalfa (CUF101, FD = 9) is a non-dormant and non-winter hardy cultivar. Data are presented by mean ± standard error. Lowercase represents significant differences at the 5% level of probability between the two cultivars

The NDT alfalfa exhibited the largely rapid regrowth condition compared with the FDT in the field after last cutting (Fig. 1a). For example, there was a significant difference in natural plant height (NPH) between FDT and NDT alfalfa cultivars in two consecutive years. The NPH of NDT alfalfa was significantly greater than that of FDT alfalfa (*P* < 0.05; Fig. 1b). However, averaged over both years, the winter survival rate of FDT alfalfa (94.67%) was about 34-fold higher than that of NDT alfalfa (2.66%; *P* < 0.05; Fig. 1c).

### Higher raffinose and amino acids in fall dormant cultivar than non-dormant cultivar under cold acclimation

**Fig. 2:**
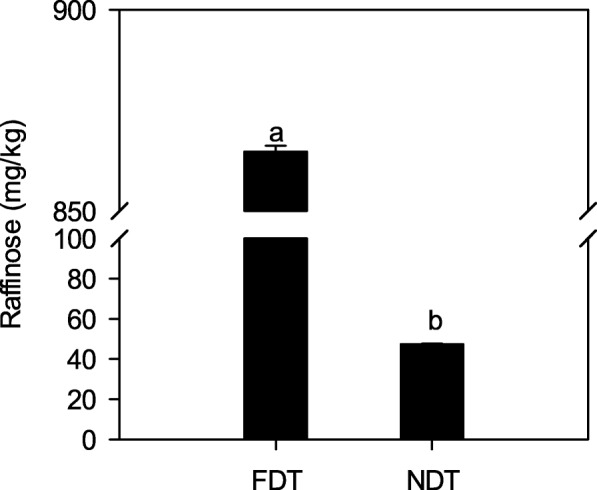
Raffinose accumulation in taproots of cold acclimating alfalfa cultivars with contrasting fall dormancy and winter hardiness. Fall dormant (FDT) alfalfa (Maverick, FD = 1) is a dormant and winter hardy cultivar, while non-dormant (NDT) alfalfa (CUF101, FD = 9) is a non-dormant and non-winter hardy cultivar. Data are presented by mean ± standard error. Lowercase represents significant differences at the 5% level of probability between the two cultivars

**Fig. 3:**
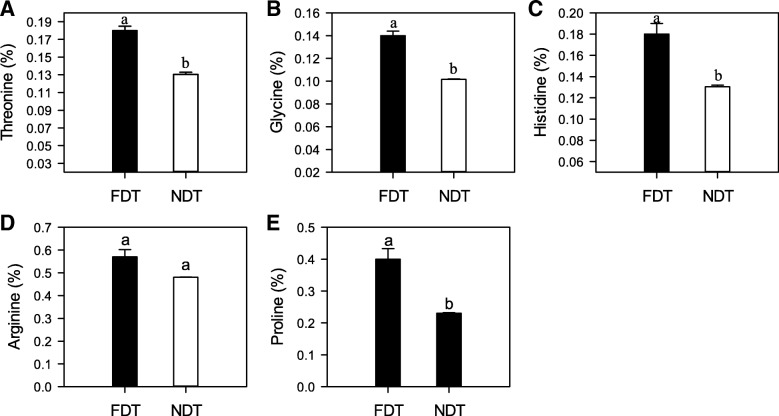
Concentrations of amino acids: Threonine (**a**), Glycine (**b**), Histidine (**c**), Arginine (**d**) and Proline (**e**) in roots of fall dormant (FDT) and non-dormant (NDT) alfalfa cultivars under cold acclimation. FDT alfalfa (Maverick, FD = 1) is a dormant and winter hardy cultivar, while NDT alfalfa (CUF101, FD = 9) is a non-dormant and non-winter hardy cultivar. Data are presented by mean ± standard error. Lowercase represents significant differences at the 5% level of probability between the two cultivars

To investigate the raffinose and amino acids accumulation in two types of alfalfa under cold acclimation, we determined the concentrations of raffinose and amino acids (Figs. 2 and 3). The raffinose concentration of FDT alfalfa roots (864.79 mg/kg) was 17.3-fold greater than that of NDT alfalfa roots (47.37 mg/kg; *P* < 0.05; Fig. 2). Among amino acids, the concentrations of threonine, glycine, histidine, and proline were significantly increased in FDT alfalfa roots (*P* < 0.05; Fig. 3a, b, c and e). Only the arginine concentration in FDT alfalfa roots was slightly higher than that in NDT alfalfa roots (Fig. 3d).

### De novo assembly and functional annotation of alfalfa root transcriptome

**Fig. 4:**
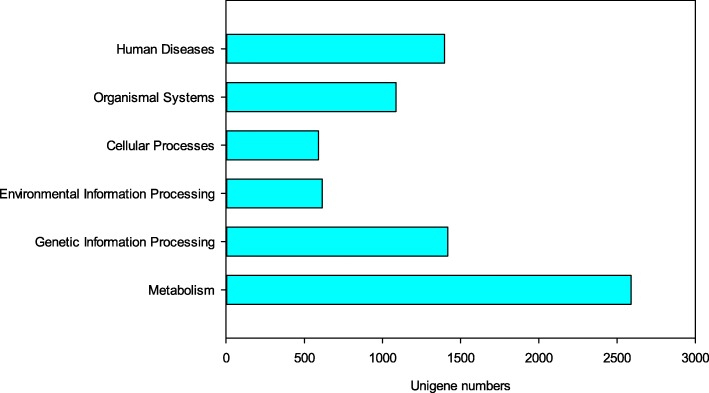
Summary of the KEGG pathways of the assembled unigenes in fall dormant and non-dormant alfalfa cultivars under cold acclimation. Blue bar represents the unigene numbers of each pathway

**Fig. 5:**
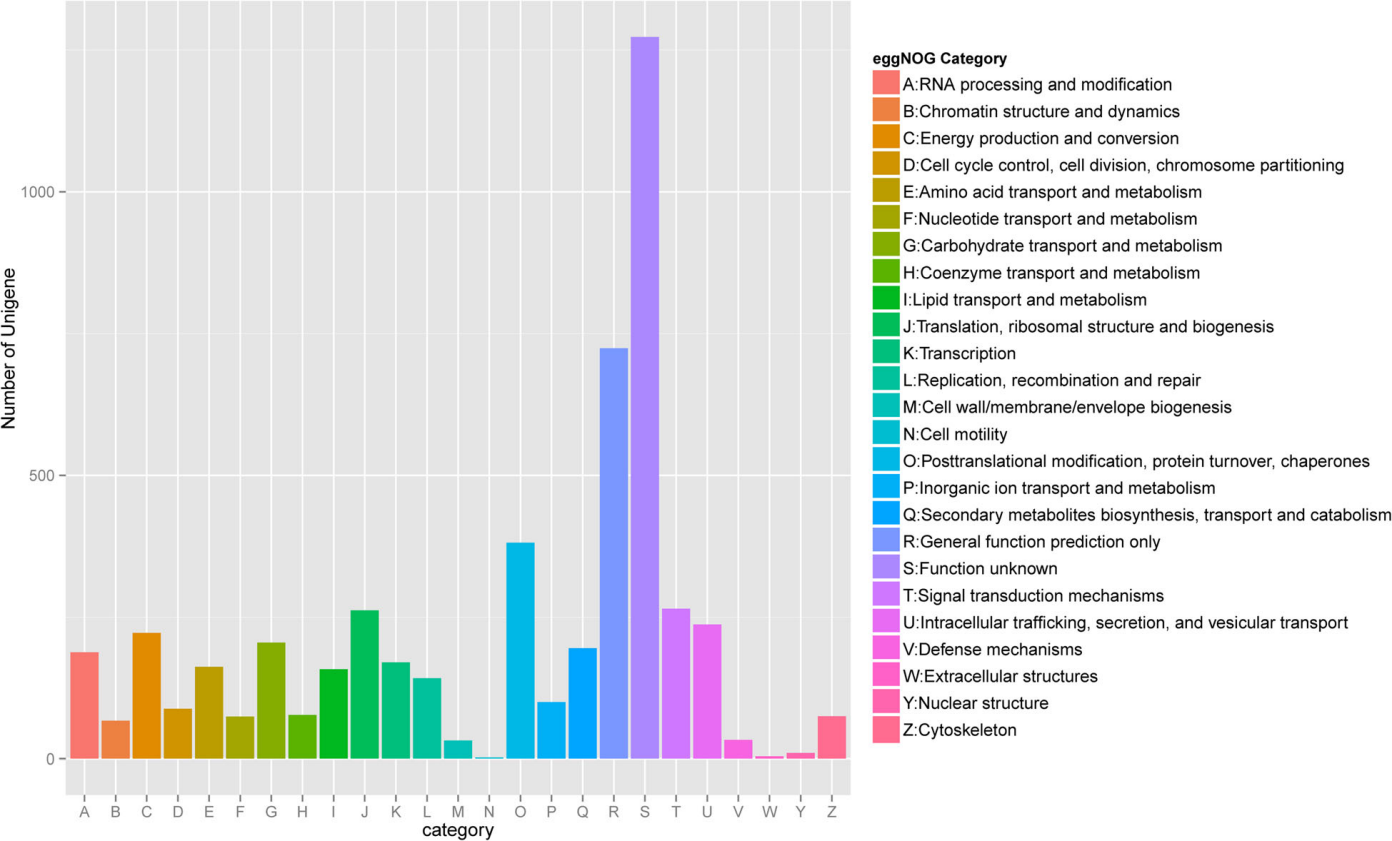
Histogram of the eggNOG (evolutionary genealogy of genes: Non-supervised Orthologous Groups) functional classification of all of the alfalfa root unigenes. Out of the 23,470 de novo assembled unigenes, 18,617 (79.32%) were annotated and grouped into 25 categories

RNA extracted from FDT and NDT alfalfa roots treated by cold acclimation was sequenced by using a next-generation sequencer. Raw reads of 90,343,369 and 138,968,136 were generated from the FDT and NDT group, respectively. After removing the adaptor sequences, ambiguous nucleotides, and low-quality sequences, a total of 167,345,864 clean reads remained for future assembly. 240,661 transcripts and 23,470 unigenes with N50 values of 992 and 2048 bp, respectively, were obtained using the Trinity software (Table 1; Additional file 1: Figure S1). Annotations were assigned to all the unigenes by BLASTx searches (*E* ≤ 10^− 5^) against five public databases (Nr, GO, Swiss-Prot, KEGG, and KOG). A total of 16,190 unigenes (68.98%) were assigned to one or more GO terms and 14,133 unigenes (60.22%) were aligned to entries in the Swiss-Prot protein sequence database (Table 2). 7696 unigenes were annotated in 321 KEGG pathways, with “metabolism pathways” (2590, 33.65%) most highly represented, including carbohydrate metabolism and amino acid metabolism, followed by “genetic information processing” (1417, 18.41%) (Fig. 4). In addition, a total of 18,617 unigenes were divided into 25 eggNOG categories (Fig. 5).

**Table 1:** A summary of the transcriptome sequencing and assembly results in the fall dormant (FDT) and non-dormant (NDT) alfalfa roots

	Sample	Total Number	Total Length (bp)	Ave Length (bp)	N50 (bp)
Raw reads	FDT	90,343,369			
	NDT	138,968,136			
Clean reads	FDT	66,297,107			
	NDT	101,048,757			
Contig	FDT+ NDT	527,916	158,278,743	299.82	427
Transcript	FDT+ NDT	240,661	163,855,683	681	992
Unigene	FDT+ NDT	23,472	34,500,666	1470	2048

Ave Length: Average length of the assembled sequence

N50: The length of the contig, transcript or unigene corresponding to the sequence, which is added to 50% of the total assembled bases when the assembled sequences are sorted from long to short

**Table 2:** Summary of the functional annotations of the assembled unigenes

Public protein database	Number of unigene hits	Percentage (%)
NR	22,968	97.86
GO	16,190	68.98
Swiss-P rot	14,133	60.22
KEGG	7696	32.79
NOG	18,617	79.32

### Exploration and GO enrichment analysis of differentially expressed genes (DEGs)

**Fig. 6:**
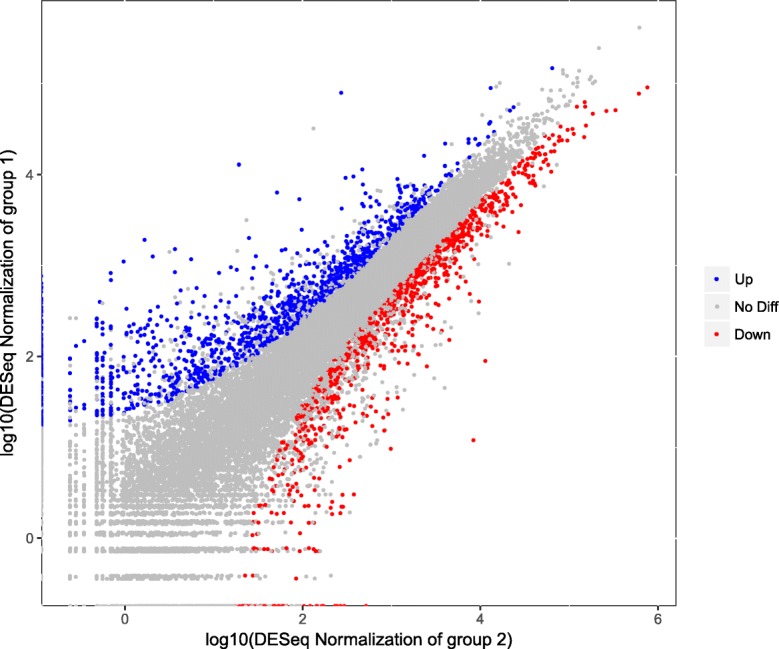
Fall dormant (FDT)-VS-non-dormant (NDT) differentially expressed genes (DEGs). DEGs were filtered using false discovery rate (FDR) ≤ 0.001 and |Log2Fold-Change| ≥ 2 as thresholds. The group 1 represents FDT alfalfa cultivar, and group 2 represents NDT alfalfa cultivar. The blue, red and gray spots represent the up-regulated, down-regulated DEGs and genes without obvious changes in FDT and NDT alfalfa cultivars under cold acclimation, respectively

Differentially expressed genes (DEGs) were identified by matching the thresholds of false discovery rate (FDR) ≤ 0.001 and |Log2Fold-Change| ≥2 as thresholds in FDT and NDT alfalfa cultivars. Totally, 2326 DEGs were obtained, including 1308 up-regulated genes and 1018 down-regulated genes (Fig. 6).

**Fig. 7:**
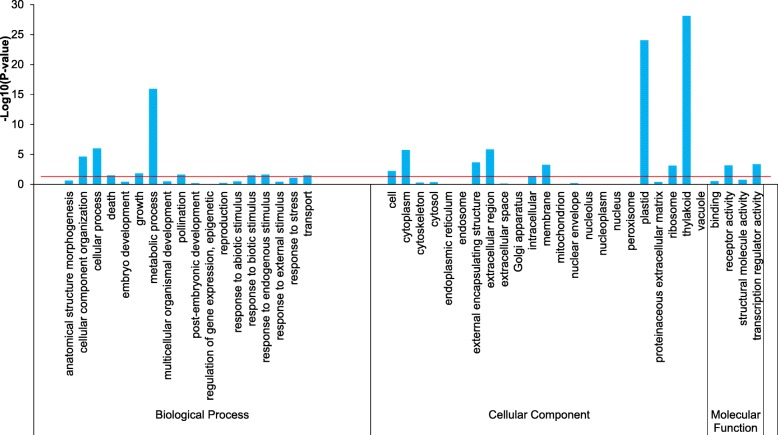
Histogram of GO Enrichment Analysis of differentially expressed genes in fall dormant (FDT) and non-dormant (NDT) alfalfa under cold acclimation. The x-axis represents the GO functional categories, grouped into Biological Process, Cellular Component and Molecular Function. The y-axis indicates the enrichment degree significance *P*-value calculated by hypergeometric distribution in each term. The red horizontal line represents *P*-value = 0.05

GO enrichment analysis was performed to explore the main functional categories of these DEGs. A total of 3 categories and 43 GO terms were identified (Fig. 7; Additional file 2: Table S1). ‘Metabolic process’ term (GO:0008152) in the biological process category was significantly enriched. Among ‘metabolic process’ term, genes for alkaline alpha galactosidase I (c92952_g2_i2) and beta-fructofuranosidase (c85655_g1_i5) related to raffinose metabolism were significantly up-regulated in FDT alfalfa, but down-regulated in NDT alfalfa. The gene encoding glutamine synthase (c66655_g1_i1) related to proline was down-regulated in FDT alfalfa, but up-regulated in NDT alfalfa. Other terms, such as ‘plastid’ and ‘thylakoid’ in the cellular component category, were also highly overrepresented in FDT alfalfa (*P*-value < 0.05; Additional file 2: Table S1).

### KEGG pathway analysis of the DEGs between FDT and NDT alfalfa under cold acclimation

In total, 593 (25.49%) of DEGs were enriched to 34 different KEGG pathways. Highly enriched pathways are shown in the Additional file 2: Table S2 The ‘carbohydrate metabolism’ pathway was the most highly enriched, followed by ‘overview’, ‘energy metabolism’, ‘amino acid metabolism’, and other pathways. More importantly, the gene (c92952_g2_i2) assigned to raffinose synthase (EC 2.4.1.82) pathway (KEGG Orthology: K06617) was significantly up-regulated in FDT alfalfa, but down-regulated in NDT alfalfa. The gene (c66655_g1_i1) assigned to arginine and proline metabolism pathways (KEGG Orthology: K01915) was significantly down-regulated in FDT alfalfa, but up-regulated in NDT alfalfa (Additional file 2: Table S3), suggesting that these pathways might participate in the metabolism of raffinose and amino acids.

### Analysis of TFs

Since TFs play a major role in regulating gene expression and plant stress responses, we performed differential expression analysis of specific TFs including CBF and ABRE-BFs during dormancy and cold acclimation (Additional file 3). A total of eight significantly differentially expressed TFs related to CBF and ABRE-BFs were identified. The most up-regulated TFs in FDT alfalfa cultivar were ABF4 (unigene c78653_g1_i1) and DREB1C (unigene c85460_g1_i2), while the most down-regulated TFs were DREB1B (unigene c89836_g1_i1).

### RNA-Seq expression validation by qRT-PCR

**Fig. 8:**
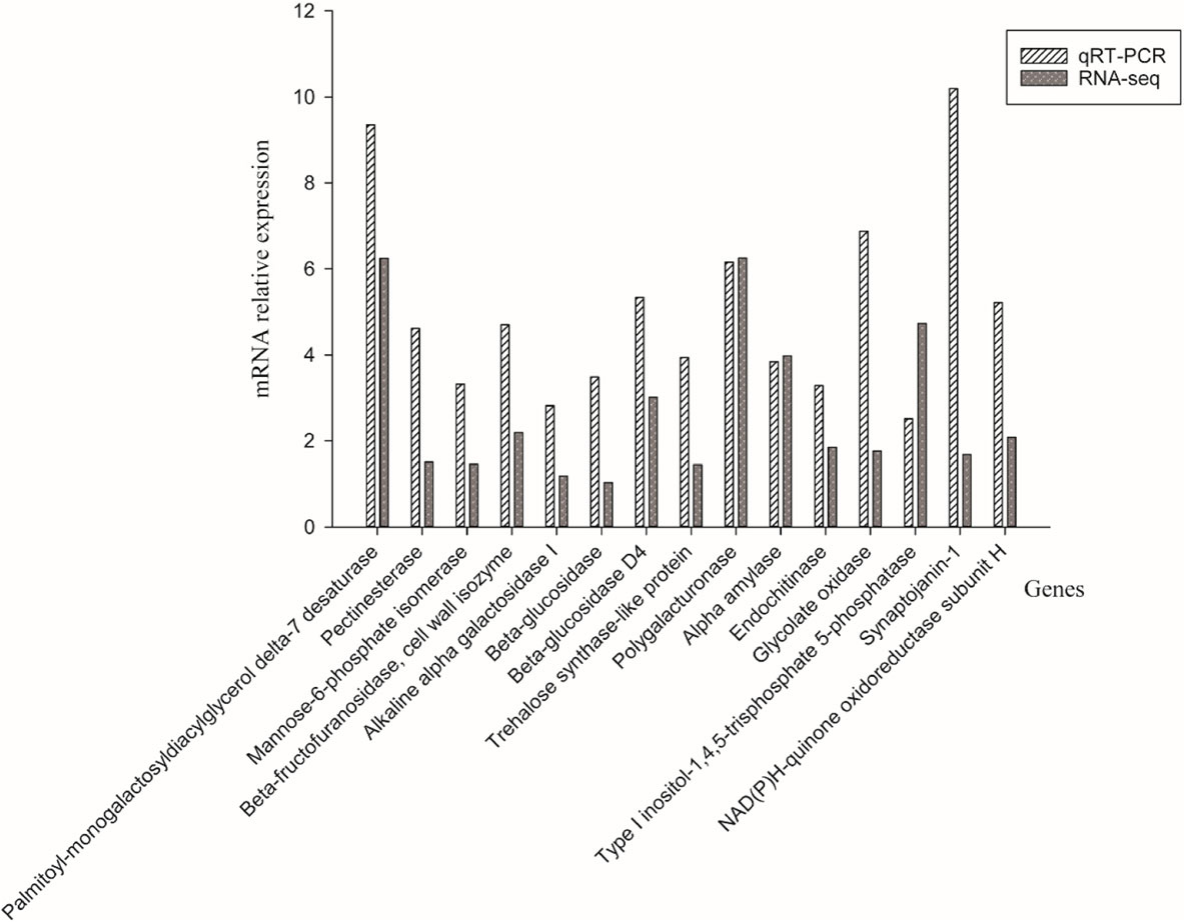
Comparison between the gene expression ratios obtained from RNA-Seq data and qRT-PCR of 15 differentially expressed genes (DEGs) in fall dormant and non-dormant alfalfa cultivars under cold acclimation. The x-axis indicates the names of 15 DEGs, and the y-axis indicates the relative gene expression levels

To further validate the reliability of our transcriptome data, 15 DEGs were randomly selected for qRT-PCR (Fig. 8). All 15 DEGs showed similar expression pattern in this analysis, suggesting that the expression of these selected genes from RNA-seq were confirmed by qRT-PCR .

## Discussion

### Characteristics of autumn regrowth and winter hardiness of fall dormant and non-dormant alfalfa

In this study, we found considerable differences in plant height after the last cutting in autumn for the two alfalfa cultivars with contrasting fall dormancy (Fig. 1). Nevertheless, it is worth mentioning that there were no notable changes in plant height between the two cultivars during growing seasons in our previous study [34]. This suggest that fall dormant alfalfa has entered the stage of fall dormancy in autumn while non dormant alfalfa has not, similar to what has been described in other perennial species, such as poplar (*Populus* spp.) [59], leafy spurge (*Euphorbia esula*) [25], blackcurrant (*Ribes nigrum*) [23], and pear (*Pyrus pyrifolia*) [4]. Fall dormant alfalfa exhibited markedly higher winter survival than non-dormant alfalfa, suggesting that fall dormant alfalfa has a stronger tolerance to cold than non-dormant alfalfa. This positive association between fall dormancy and winter survival has also been reported in other studies [33, 35, 41]. They recommended that fall dormancy be used as a choice indicator for improving alfalfa winter hardiness [6]. However, this approach would have significant negative effects on forage yield potential because fall dormant alfalfa cultivars regrow slowly after the last harvest in autumn and produce lower forage yield than non-dormant alfalfa [44, 53, 55]. Based on our findings, we can speculate that the two cultivars have significant differences in accumulation of raffinose, etc. as a result of shorter day length and lower temperature during late autumn.

### Higher raffinose accumulation and up-regulated genes associated with raffinose metabolism in fall dormant alfalfa than non-dormant alfalfa under cold acclimation

Raffinose plays an important role in adaptation to stress environments for plants [19]. In this study, our results clearly showed that raffinose concentration in fall dormant alfalfa roots is markedly higher than that in non-dormant alfalfa roots (Fig. 2), consistent with what has been observed by Cunningham et al. [14]. This indicates that fall dormant alfalfa can accumulate more raffinose than non-dormant alfalfa under cold acclimation. The accumulation of raffinose is closely associated with alfalfa winter survival in overwintering tissues [48]. It is likely that the elevated level of raffinose contributes to enhancing tolerance to freezing under cold acclimation in fall dormant alfalfa [8]. Therefore, it is reasonable to infer that raffinose was at least partially responsible for the observed increased winter survival in fall dormant alfalfa (Fig. 1c). The possible explanations for the physiological effect of raffinose accumulation on winter survival are as follows. On one hand, the accumulation of raffinose in the roots as general cryoprotectants preferentially induced in the FDT line primarily by the cold acclimating conditions may contribute to the observed differences in winter survival between the lines [18, 29, 43]. On the other hand, raffinose transports into the plastids across the tonoplast during the intracellular re-distribution process, which is indispensable in sustaining the compatible carbohydrate homeostasis under cold acclimation [24]. However, in the non-dormant alfalfa, raffinose accumulation may be insufficient to protect against cold damage.

It is likely that raffinose synthase (RS, EC 2.4.1.82) is essential for raffinose biosynthesis in plants [48]. Raffinose accumulation in roots of fall dormant alfalfa under cold acclimation indicated that some genes encoding RS may be involved in this process. Previous transcription profiles comparing fall dormant and non-dormant alfalfa leaf tissues revealed several differentially expressed genes [61]. In this study, we confirmed the correlation between fall dormancy and raffinose metabolism under cold acclimation, and identified DEGs between FDT and NDT lines following cold acclimation are enriched in ‘metabolic process’ term (GO:0008152; Fig. 7). More importantly, our data clearly documented that the expression of a candidate gene (e.g. c92952_g2_i2) related to RS pathway was significantly up-regulated in fall dormant alfalfa, but down-regulated in non-dormant alfalfa (Additional file 2: Table S3). Similarly, the up-regulation of genes related to RS were also detected in rice during cold acclimation [47]. This suggests that the differential expression of the candidate genes related to RS probably contributes to raffinose accumulation in fall dormant alfalfa roots under cold acclimation. Overall, this integrated analysis of raffinose accumulation and RS transcript levels suggests fall dormancy and raffinose accumulation is correlated to winter survival in alfalfa. Interestingly, specific TFs including CBF and ABRE-BFs involved in control of genes during dormancy and cold acclimation were differentially expressed between fall dormant and non-dormant alfalfa cultivars under cold acclimation. For example, ABF4 (unigene c78653_g1_i1) and DREB1C (unigene c85460_g1_i2) were the most up-regulated TFs in fall dormant alfalfa cultivar, but were down-regulated in the non-dormant cultivar. It is likely that the fall dormant lines are able to induce cold acclimation TFs to increase winter survival, while the non-dormant lines are not.

### Amino acid accumulation and changes of genes related to amino acid metabolism in fall dormant and non-dormant alfalfa under cold acclimation

Proline, one of the most recognized osmoprotectants, is a major solute which accumulates in plants in response to several environmental stresses, such as low temperature [27]. The increase of its accumulation during cold acclimation occurs, e.g. in *Brassica napus* [28], *Chrysanthemum dichrum* [11], and *Poncirus trifoliate* [42]. Also, transgenic tobacco (*Nicotiana tabacum*) over-expressing crucial genes for proline synthesis showed greater cold tolerance than the wild type [39]. In the present study, the accumulation of proline in fall dormant alfalfa roots was notably higher than that in non-dormant alfalfa (Fig. 3), similar to what has been observed by Szabados and Savoure, who found that there was a high proline accumulation in alfalfa roots during cold acclimation [50]. We speculate that the increase of proline accumulation may be triggered in fall dormant alfalfa under cold acclimation. Furthermore, based on the transcriptomic profile, the gene for glutamine synthetase (c66655_g1_i1) related to proline was down-regulated in fall dormant alfalfa, but up-regulated in non-dormant alfalfa. This indicates that the glutamine synthesis decreases, and glutamic acid increases relatively in fall dormant alfalfa under cold acclimation. Plants produce proline mainly through the glutamic acid process when they are exposed to cold acclimation. Therefore, fall dormant alfalfa can use more glutamic acid to produce proline than non-dormant alfalfa under cold acclimation. Interestingly, there is a similarity of arginine accumulation patterns to proline. In fall dormant alfalfa roots under cold acclimation, arginine may serve as a chelator of nitrate to prevent damage to membranes and ice formation [30], while this capacity is not available in non-dormant alfalfa. Moreover, the increased arginine may bind excess ammonia under cold stress to adapt to the cold environment in fall dormant alfalfa [9].

Similar to proline accumulation, the accumulation of threonine, glycine, and histidine also significantly increased in fall dormant alfalfa roots compared with non-dormant alfalfa roots (Fig. 3). The prominence of threonine observed in our study confirms data from Paul et al., who indicated that threonine plays a vital role in winter hardiness of fall dormant alfalfa [40]. This result is also consistent with the potential association of glycine and histidine with the cold acclimation process, as suggested in alfalfa [16] and *Pinus radiate* [48]. This implies that fall dormant alfalfa grows slowly or stops growing after the last cutting in autumn and accumulates more amino acids (e.g. proline, threonine, and histidine) in preparation for overwintering than non-dormant alfalfa.

## Conclusions

In summary, our study indicates the correlation between these changes in gene expression and accumulation of specific sugars are correlated with differences in fall growth, dormancy, and winter survival in alfalfa. Fall dormant alfalfa drastically increased raffinose and amino acid accumulation under cold acclimation. Raffinose-associated and amino acid-associated genes involved in metabolic pathways were expressed at higher levels in fall dormant alfalfa than non-dormant alfalfa under cold acclimation. The changes in gene expression are consistent with observed increases in raffinose and amino acids concentration in fall dormant alfalfa under cold acclimation.

## Methods

### Plant material and growth conditions

Two alfalfa cultivars, Maverick (FD = 1.0) and CUF101 (FD = 9.0), were used in the experiments. These two cultivars were selected because they show minimal variation across environments [6]. Plants were initially established in a greenhouse in Wuyuan County, midwestern Inner Mongolia (40°46′N, 107°35′E; elevation 1102.7 m), China, to ensure uniformity and to minimize uncontrolled sources of stress. On 4 April 2014, alfalfa seeds were sown into standard cultivation pots (top diameter 4 cm, bottom diameter 1.5 cm, height 20.5 cm) that were deep enough to allow root development, filled with a nutrient soil matrix (a commercial nursery substrate, provided by Xinyuan Turf Production Base, China). Each pot contained two or three seeds with 150 pots (30 plants per replication, five replications) per cultivar. All alfalfa seeds were inoculated with a commercial inoculant of *Sinorhizobium meliloti* Dang before planting. Seedlings were thinned by hand to one plant per pot 3 weeks after germinating. The temperature of the greenhouse was 17 °C to 26 °C and plants were grown following a natural photoperiod with approximately 10 h of daylight. All plants were watered equally every 3 days. The volume of water, which is determined by seedlings growth states, is low in the first 2 weeks but high in the last 6 weeks. Weeds were timely removed by hand. Fortunately, no insects were found during seedling cultivation.

After 8 weeks of growth in the greenhouse, all alfalfa plants were transferred to an experimental field on 11 June 2014 near Hohhot City (Latitude 40°83′N; Longitude 111°73′E; Elevation 1040 m), Inner Mongolia, China. The soil was a sandy loam and contained (0–20 cm depth) 11.0 g/kg of organic matter (OM), 0.66 g/kg of total nitrogen (TN), 110.0 mg/kg of available nitrogen (AN), 10.0 mg/kg of available phosphorus (AP), 190.0 mg/kg of available potassium (AK) and 1.11 g/kg of total salt with pH 8.32 (1:5 in water). Each plot was 9.0 m long and consisted of four rows and 30 plants of alfalfa per row with 0.6 m row spacing and 0.3 m plant spacing. Each row represented one cultivar. The plot was designed as a randomized complete block with five replicates. The alfalfa cultivar Zhongmu No.1 was sowed as a protective plant around the plot. Plants were kept well-watered and urea as a fertilizer (a commercial fertilizer, N content was 46.2% and rate of application was 150 kg/hectare, provided by Shandong Mingshui Dahua Ltd., China) was applied on 10 July 2014. Plants were defoliated twice at the development of 10% of the flowers during the summer, on 5 July 2014 and on 6 August 2014, respectively. The last defoliation in the fall was performed on the 26 August 2014, a date that was chosen based on our previous experiences and as suggested by another study [6].

### Physiological determination

Plant height was measured 21 days after the last defoliation in the fall, following the study of Barnes et al. [6]. According to the natural temperature changes in the field, root samples were collected on 5 December 2014. For each cultivar, the roots of three biological replicates were dug out and washed free from soil under a stream of cold water. Approximately the first 20-cm of taproots were sampled in a randomly selected plant from the plot. Root samples for raffinose and amino acid analysis were freeze-dried on a cryofreeze-dryer (Heto, Thermo Fisher Scientific Heto, USA) and were ground to pass a 1-mm screen and stored at − 20 °C. Root samples for RNA-Seq and qRT-PCR were cut into small segments (about 4–5 mm), frozen immediately in liquid nitrogen and then stored at − 80 °C.

Raffinose was measured by HPLC (Waters Scientific, Milford, MA) on a Sugar-Pak column (6.5×300 mm) eluted isocratically with water containing 0.13 mM EDTA (Ca^2+^, Na^+^) at 85 °C and quantitated with a differential refractometer. Amino acid content was measured by the Amino acid automatic analyzer (A300, Amino acid Analyzer, MembraPure GmbH). In the statistical analysis, we conducted an analysis of ANOVA and post hoc test using Tukey’s.

### cDNA library construction and sequencing

Total RNA was extracted from six samples (three biological replicates per cultivar) using the QIAGEN RNeasy Plant Mini kit (Qiagen) according to the manufacturer’s protocol. Contaminating genomic DNA was removed from each RNA sample using DNase I. RNA samples were quantified using Quant-iT Pico-Green RNA Reagent (https://www.thermofisher.com/cn/zh/home/brands/invitrogen.html), and RNA integrity was checked with the RNA6000 Nano Assay using the Agilent 2100 Bioanalyzer (Agilent Technologies). The RNA samples were used to construct the root-specific cDNA libraries for RNA sequencing and transcriptome analysis. According to the manufacturer Illumina’s instructions, mRNAs were purified using poly-A oligonucleotide-attached magnetic beads and then fragmented. The first- and second-strand cDNAs were synthesized and end repaired. Adaptors were ligated after adenylation at the 3′ ends. After gel purification, cDNA templates were enriched by PCR. The cDNA library was quantified using Quant-iT PicoGreen dsDNA Assay Kit (https://www.thermofisher.com/cn/zh/home/brands/invitrogen.html) and by Quantifluor-ST fluorometer (Promega Technologies). The cDNA library was validated using an Agilent High Sensitivity DNA Kit on the Agilent 2100 Bioanalyzer (Agilent Technologies). The libraries were then sequenced using the Illumina NextSeq 500 by Personalbio Technology Co. Ltd., Shanghai, China. The sequencing data has all been archived in the NCBI Sequence Read Archive (SRA) database under accession number PRJNA489768.

### De novo assembly of sequencing reads and sequencing clustering

Following cDNA library sequencing, high-quality clean reads were selected from the raw reads of each library, following removal of reads containing adaptor sequences, reads with an N (unknown bases in a read) percentage higher than 5% and low-quality reads (> 50% of the bases with a quality score Q-value ≤5) using Perl scripts. Transcriptome de novo assembly was carried out with the short-reads assembly programme Trinity [22]. We first combined reads with a certain length of overlap to form contigs with a zero N value (no unknown bases). The K-mer value of the overlapping sequence was 25. Then, the reads were mapped back to contigs with paired-end reads. This approach detected contigs from the same transcript as well as the distances between these contigs. Next, we used Trinity to connect the contigs to form scaffolds using N to represent unknown sequences between each contig pair. Finally, unigenes were generated with zero N values in the sequence that could not be extended on either end. In the final step, a BLASTX alignment (e value < 0.00001) was performed between the recovered unigenes and protein databases such as nr, Swiss-Prot, KEGG (Kyoto Encyclopedia of Genes and Genomes) and COG (Clusters of Orthologous Groups), and the best aligning results were used to determine the sequence direction of the unigenes. If the results from different databases conflicted with one another, a priority order of nr, Swiss-Prot, KEGG and COG was followed when deciding the unigene sequence direction. When a unigene could not be aligned to any of the databases, the programme ESTScan was used to determine its sequence direction. For unigenes with verified sequence directions, we provided their sequences in the 5′ to 3′ orientation; for those without direction, we provided their sequences as determined by the assembly software.

### Quantification of gene expression levels and differential expression analysis

For gene expression analysis, the expression level of each gene in each library was calculated by quantifying the number of Illumina reads that mapped to each of the LAGI 1.0 sequences using the Bowtie program with default parameters [32].The raw gene expression counts were normalized using the RPKM (Reads per kb per million reads) method with the formula RPKM = 10^6^C/10^−3^NL, where C is the number of reads that uniquely align to a unigene, N is the total number of reads that uniquely align to all unigenes, and L is the base number in the Coding sequence (CDS) of a unigene [37]. The gene expression data is shown in the Additional file 4. Genes exhibiting differential expression were identified using the DESeq program to perform pairwise differential expression analysis [1]. We compared the transcriptome profiles of FDT and NDT alfalfa under cold acclimation to detect DEGs between the two cultivars using the statistical method FDR (False discovery rate) and the ratio of RPKMs for the two samples (FDR ≤ 0.001 and log2 ratio > 2). The raw data that shows differential expression between FDT and NDT alfalfa is shown in Additional file 5. DEGs were then used to carry out GO (gene ontology) functional and KEGG Pathways analysis.

### Unigene functional annotation and classification

The individual unigenes were analysed by mining the protein databases nr, Swiss-Prot, KEGG and COG (e value < 0.00001) with the BLASTX algorithm (http://www.ncbi.nlm.nih.gov/), in order to retrieve the functionally annotated proteins showing the highest sequence similarity to the unigenes on our list. The COG database was used to predict and to classify possible functions of our unigenes. The KEGG pathways database was used to annotate inner-cell metabolic pathway and to determine the potential complex biological behaviors of the genes on our list. The Blast2GO programme was used to interpret the GO functional annotations derived from the molecular function, cellular location and biological processes of all our unigenes [12].

### Analysis of transcription factors

Specific transcription factors, CBF and ABRE-BFs both of which are involved in cold acclimation processes, were identified using BLASTX in NCBI. Sequences of all unigenes were blasted with that of genes related to CBF and ABRE-BFs from *Arabidopsis thaliana*. A total of 104 TFs were identified, then the differentially expressed TFs were picked out from DEGs in FDT and NDT cultivars by fold change> 2 or < 0.5 and *P* < 0.05.

### Quantitative real-time PCR analysis (qRT-PCR)

RNA was extracted from the roots of FDT and NDT alfalfa under cold acclimation. cDNA was synthesized from 2 μg of RNase-free, DNase I-treated total RNA with 500 ng of 18mer oligo-dT primers and M-MLV reverse transcriptase (Promega). The primers for qRT-PCR were designed using the sequences determined for the differentially expressed unigenes using the program Primer 5.0 (Additional file 2: Table S4). Primers were also tested to ensure that primer products resulted in a single discrete band amplification. They are designed to amplify a region over a non-conserved region. The *Medicago sativa* putative glyceraldehyde-3-phosphate dehydrogenase (GAPDH) gene, a putative housekeeping gene, was used as an endogenous control [62]. Three technical replicates were included for each sample and a negative control was included in each run (control lacking a cDNA template). Quantitative RT-PCR was performed using the CFX96 real-time PCR detection system (Bio-Rad), a 25-μl reaction system and the SYBR Premix Ex Taq Kit (TaKaRa Corp. Beijing, China) according to the manufacturer’s protocol. The following cycling program was used: 95 °C for 60 s, followed by 40 cycles of 10 s at 95 °C, 30 s at 55 °C and 30 s at 72 °C. All products were subjected to melting curve analysis between 55 °C and 95 °C to determine the specificity of the PCR reaction. The relative quantitative method (^∆∆^Ct) was used to evaluate the quantitative variation.

## Additional files

Additional file 1: **Figure S1.** Size distribution of the contigs, transcripts and unigenes generated by de novo assembly. (A) Size distribution of contigs. The x-axis represents contig size, and the y-axis represents numbers of contigs of a certain length. (B) Size distribution of transcripts. The x-axis represents transcript size, and the y-axis represents the number of transcripts with a certain length. (C) Size distribution of unigenes. The x-axis represents unigene size, and the y-axis represents the number of unigenes with a certain length (DOCX 28 kb)

Additional file 2: **Table S1.** Table S1 Enrichment GO terms of the differentially expressed genes corresponding to biological process, cellular component and molecular functions in fall dormant (FDT) and non-dormant (NDT) alfalfa cultivars under cold acclimation. **Table S2.** Top 20 highly enriched KEGG pathways of the differentially expressed genes between fall dormant and non-dormant alfalfa cultivars under cold acclimation. **Table S3.** KEGG pathway information of gene (c92952_g2_i2) and gene (c66655_g1_i1) in fall dormant (FDT) and non-dormant (NDT) alfalfa cultivars under cold acclimation. **Table S4.** Primer pairs (F, forward; R, reverse) of the fifteen genes of interest and a reference gene used in qRT-PCR analysis of the relative abundance of genes in root tissue of alfalfa in response to cold acclimation (DOCX 23 kb)

Additional file 3: Identification of specific transcription factors related to CBF/ABRE-BFs and list of Differentially Expressed transcription factors (XLSB 18 kb)

Additional file 4: The raw data of gene expression obtained from RNA-Seq between fall dormant and non-dormant alfalfa (XLSX 6034 kb)

Additional file 5: The raw data that shows differential expression between fall dormant and non-dormant alfalfa under cold acclimation (XLSX 400 kb)
