# Supplementary material for: How fall dormancy benefits alfalfa winter-survival? Physiologic and transcriptomic analyses of dormancy process
Source: BMC Plant Biol. 2019 May 20;19:205. doi: 10.1186/s12870-019-1773-3 (PMC6528297; doi:10.1186/s12870-019-1773-3)
Supplement: Supplementary file 1 — Figure S1. Size distribution of the contigs, transcripts and unigenes generated by de novo assembly. (A) Size distribution of contigs. The x-axis represents contig size, and the y-axis represents numbers of contigs of a certain length. (B) Size distribution of transcripts. The x-axis represents transcript size, and the y-axis represents the number of transcripts with a certain length. (C) Size distribution of unigenes. The x-axis represents unigene size, and the y-axis represents the number of unigenes with a certain length (DOCX 28 kb) [file 12870_2019_1773_MOESM1_ESM.docx]

A

B

C

**Figure S1.** Size distribution of the contigs, transcripts and unigenes generated by *de novo* assembly. (A) Size distribution of contigs. The x-axis represents contig size, and the y-axis represents numbers of contigs of a certain length. (B) Size distribution of transcripts. The x-axis represents transcript size, and the y-axis represents the number of transcripts with a certain length. (C) Size distribution of unigenes. The x-axis represents unigene size, and the y-axis represents the number of unigenes with a certain length.
