# Supplementary material for: How fall dormancy benefits alfalfa winter-survival? Physiologic and transcriptomic analyses of dormancy process
Source: BMC Plant Biol. 2019 May 20;19:205. doi: 10.1186/s12870-019-1773-3 (PMC6528297; doi:10.1186/s12870-019-1773-3)
Supplement: Supplementary file 2 — Table S1. Table S1 Enrichment GO terms of the differentially expressed genes corresponding to biological process, cellular component and molecular functions in fall dormant (FDT) and non-dormant (NDT) alfalfa cultivars under cold acclimation. Table S2. Top 20 highly enriched KEGG pathways of the differentially expressed genes between fall dormant and non-dormant alfalfa cultivars under cold acclimation. Table S3. KEGG pathway information of gene (c92952_g2_i2) and gene (c66655_g1_i1) in fall dormant (FDT) and non-dormant (NDT) alfalfa cultivars under cold acclimation. Table S4. Primer pairs (F, forward; R, reverse) of the fifteen genes of interest and a reference gene used in qRT-PCR analysis of the relative abundance of genes in root tissue of alfalfa in response to cold acclimation (DOCX 23 kb) [file 12870_2019_1773_MOESM2_ESM.docx]

Table S1 Enrichment GO terms of the differentially expressed genes corresponding to biological process, cellular component and molecular functions in fall dormant (FDT) and non-dormant (NDT) alfalfa cultivars under cold acclimation.

| GO term ID | Term type | Term description | -LOG10(P-value) |
| --- | --- | --- | --- |
| GO:0009653 | P | anatomical structure morphogenesis | 0.605855937 |
| GO:0016043 | P | cellular component organization | 4.598980668 |
| GO:0009987 | P | cellular process | 5.972426982 |
| GO:0016265 | P | death | 1.473579946 |
| GO:0009790 | P | embryo development | 0.425499369 |
| GO:0040007 | P | growth | 1.832590203 |
| GO:0008152 | P | metabolic process | 15.9366246 |
| GO:0007275 | P | multicellular organismal development | 0.475520792 |
| GO:0009856 | P | pollination | 1.603795595 |
| GO:0009791 | P | post-embryonic development | 0.179036533 |
| GO:0040029 | P | regulation of gene expression, epigenetic | 0.021569208 |
| GO:0000003 | P | reproduction | 0.212177575 |
| GO:0009628 | P | response to abiotic stimulus | 0.469542876 |
| GO:0009607 | P | response to biotic stimulus | 1.49200914 |
| GO:0009719 | P | response to endogenous stimulus | 1.616363054 |
| GO:0009605 | P | response to external stimulus | 0.42596769 |
| GO:0006950 | P | response to stress | 1.085611776 |
| GO:0003700 | P | sequence-specific DNA binding transcription factor activity | 0.141797675 |
| GO:0006810 | P | transport | 1.478084023 |
| GO:0005575 | C | cellular_component | 0.111068867 |
| GO:0005623 | C | cell | 2.216211152 |
| GO:0005737 | C | cytoplasm | 5.708362352 |
| GO:0005856 | C | cytoskeleton | 0.283453543 |
| GO:0005829 | C | cytosol | 0.361724349 |
| GO:0005783 | C | endoplasmic reticulum | 0.016859104 |
| GO:0005768 | C | endosome | 0.030626447 |
| GO:0030312 | C | external encapsulating structure | 3.66241576 |
| GO:0005576 | C | extracellular region | 5.828021524 |
| GO:0005794 | C | Golgi apparatus | 0.004001992 |
| GO:0005622 | C | intracellular | 1.36630042 |
| GO:0016020 | C | membrane | 3.267617982 |
| GO:0005739 | C | mitochondrion | 0.024641637 |
| GO:0005730 | C | nucleolus | 0.055624829 |
| GO:0005634 | C | nucleus | 0.00025657 |
| GO:0005777 | C | peroxisome | 0.04449115 |
| GO:0009536 | C | plastid | 24.02924004 |
| GO:0005840 | C | ribosome | 3.117759639 |
| GO:0009579 | C | thylakoid | 28.12135925 |
| GO:0005773 | C | vacuole | 0.000332189 |
| GO:0003674 | F | molecular_function | 0.515481644 |
| GO:0005488 | F | binding | 3.155391934 |
| GO:0004872 | F | receptor activity | 0.74562113 |
| GO:0005198 | F | structural molecule activity | 3.337557052 |

P - Biological Process; C - Cellular Component; F - Molecular Function.

Table S2 Top 20 highly enriched KEGG pathways of the differentially expressed genes between fall dormant and non-dormant alfalfa cultivars under cold acclimation.

| Order | KEGG pathways | Differentially expressed gene numbers |
| --- | --- | --- |
| 1 | Carbohydrate metabolism | 87 |
| 2 | Overview | 67 |
| 3 | Energy metabolism | 63 |
| 4 | Amino acid metabolism | 50 |
| 5 | Biosynthesis of other secondary metabolites | 42 |
| 6 | Lipid metabolism | 36 |
| 7 | Translation | 35 |
| 8 | Signal transduction | 35 |
| 9 | Infectious diseases | 31 |
| 10 | Metabolism of other amino acids | 20 |
| 11 | Cell growth and death | 19 |
| 12 | Metabolism of cofactors and vitamins | 18 |
| 13 | Replication and repair | 16 |
| 14 | Xenobiotics biodegradation and metabolism | 14 |
| 15 | Cancers | 14 |
| 16 | Metabolism of terpenoids and polyketides | 13 |
| 17 | Transport and catabolism | 12 |
| 18 | Endocrine system | 12 |
| 19 | Environmental adaptation | 12 |
| 20 | Folding, sorting and degradation | 10 |

Table S3. KEGG pathway information of gene (c92952_g2_i2) and gene (c66655_g1_i1) in fall dormant (FDT) and non-dormant (NDT) alfalfa cultivars under cold acclimation.

|  | | KEGG pathway | KEGG Orthology | FDT vs NDT alfalfa | |
| --- | --- | --- | --- | --- | --- |
|  |  |  |  | Up-regulated | Down-regulated |
| Gene ID | c92952_g2_i2 | raffinose synthase [EC:2.4.1.82] | K06617 | Up-regulated | Down-regulated |
|  | c66655_g1_i1 | glutamine synthetase [EC:6.3.1.2] | K01915 | Down-regulated | Up-regulated |

Table S4 Primer pairs (F, forward; R, reverse) of the fifteen genes of interest and a reference gene used in qRT-PCR analysis of the relative abundance of genes in root tissue of alfalfa in response to cold acclimation.

| Gene name | Gene ID or GenBank Accession No. | Primer sequence | Predicted fragment size (bp) | Annealing temp. (°C) |
| --- | --- | --- | --- | --- |
| Palmitoyl-monogalactosyldiacylglycerol delta-7 desaturase | c84245_g1_i1 | F: 5’ CTTGTGTGCCCAGAGACGAT 3’ | 207 | 60.04 |
|  |  | R: 5’ ATCACCGACACACCCATCAC 3’ |  | 60.04 |
| Pectinesterase | c92985_g1_i3 | F: 5’ TGGATCGGTTCTTCCTTGCG 3’ | 105 | 60.39 |
|  |  | R: 5’ TTCATCTTTGGAAACGCCGC 3’ |  | 59.76 |
| Mannose-6-phosphate isomerase | c93507_g1_i1 | F: 5’ CTGTTCCAGGTCCGTCCATC 3’ | 136 | 60.11 |
|  |  | R: 5’ TCACTCGCGACGCTAATCTC 3’ |  | 59.97 |
| Beta-fructofuranosidase | c85655_g1_i5 | F: 5’ CCACCAAAACTCTCCACCACT 3’ | 145 | 60.13 |
|  |  | R: 5’ TCATGTGCAGCGATCAAAGC 3’ |  | 59.55 |
| Alkaline alpha galactosidase I | c92952_g2_i2 | F: 5’ TTCACTCCGCCCCAATAACC 3’ | 117 | 60.03 |
|  |  | R: 5’ AGAGCACAACGAACCGACAT 3’ |  | 59.97 |
| Beta-glucosidase | c95333_g1_i3 | F: 5’ AATGGGAGGCTATGATGCAGG 3’ | 179 | 59.92 |
|  |  | R: 5’ ACCCCTGTTGCTTGACCTTG 3’ |  | 60.47 |
| Beta-glucosidase D4 | c93868_g1_i1 | F: 5’ GGTGGTGATTCTGGAACCGA 3’ | 151 | 59.68 |
|  |  | R: 5’ AGAGCGGCACATTCCAGTTA 3’ |  | 59.39 |
| Trehalose synthase-like protein | c82669_g1_i1 | F: 5’ TTGGTTCGTTATGCGAGGGT 3’ | 158 | 59.68 |
|  |  | R: 5’ ACGAACCACACGTCAACCTT 3’ |  | 60.11 |
| Polygalacturonase | c26298_g1_i1 | F: 5’ TATCTCCGTTTGGTTGGGCG 3’ | 121 | 60.39 |
|  |  | R: 5’ CTCTTCTGTTGCTTTGCCCC 3’ |  | 59.40 |
| Alpha amylase | c88543_g1_i1 | F: 5’ ACCTTGAACAGCAGCACCTAA 3’ | 173 | 59.86 |
|  |  | R: 5’ ATTTCGCGGTTGGAGAGCTT 3’ |  | 60.32 |
| Endochitinase | c78640_g1_i1 | F: 5’ TCCTCTTCCGCATTCAAGCC 3’ | 153 | 60.39 |
|  |  | R: 5’ TTTTGGATGACGCCCCAGTC 3’ |  | 60.61 |
| Glycolate oxidase | c88179_g1_i5 | F: 5’ TCGGGGTGAGCCATCTTTTG 3’ | 128 | 60.32 |
|  |  | R: 5’ ATTCTGTTCAGGCCGCGAAT 3’ |  | 60.39 |
| Type I inositol-1,4,5-trisphosphate 5-phosphatase | c87352_g1_i5 | F: 5’ TGCCGAAAGCATAGTTGCCA 3’ | 249 | 60.61 |
|  |  | R: 5’ AGGCCAACGGGTTTTGTTCA 3’ |  | 60.68 |
| Synaptojanin-1 | c90951_g3_i3 | F: 5’ GCAGAAGGTGGAGTTTTTGAGG 3’ | 114 | 59.71 |
|  |  | R: 5’ TCTAGTTGGAAGGCCACCAC 3’ |  | 59.31 |
| NAD(P)H-quinone oxidoreductase subunit H | c91603_g5_i1 | F: 5’ GGTTTCAACGAATCGCACGTA 3’ | 103 | 59.54 |
|  |  | R: 5’ TCCTTTTTGGGTGGTCTACGA 3’ |  | 58.95 |
| Reference gene （GAPDH） | GQ398120.1 | F: 5’ GGCAAGCTCAACGGTATTGC 3’ | 125 | 60.18 |
|  |  | R: 5’ GCTGCACTGTCTCTGAAAGC 3’ |  | 59.48 |
